# Health risk behaviors among medical and nursing students of Lumbini Medical College, Nepal: A cross‐sectional study

**DOI:** 10.1002/hsr2.70140

**Published:** 2024-10-16

**Authors:** Samata Nepal, Alok Atreya, Kishor Adhikari, Bhumika Acharya, Ritesh G. Menezes, Laxmi Prasad Sapkota

**Affiliations:** ^1^ Department of Community Medicine Lumbini Medical College Palpa Nepal; ^2^ Department of Forensic Medicine Lumbini Medical College Palpa Nepal; ^3^ Department of Public Health Chitwan Medical College Chitwan Nepal; ^4^ Medical Student Lumbini Medical College Palpa Nepal; ^5^ Forensic Medicine Division, Department of Pathology College of Medicine, Imam Abdulrahman Bin Faisal University Dammam Saudi Arabia

**Keywords:** alcohol drinking, mental health, physical activity, risk‐taking, sleep, substance use

## Abstract

**Background and Aims:**

Healthcare students are expected to lead healthy lives yet they may engage in health risk behaviors (HRBs) like physical inactivity, poor diet, and substance use. These behaviors can have negative consequences for both the individual's health and well‐being, as well as their ability to perform their future roles as healthcare providers. This study aimed to assess the prevalence of HRBs and associated factors among medical and nursing students at Lumbini Medical College, Nepal.

**Methods:**

A cross‐sectional study was conducted among 412 undergraduate healthcare students using a self‐administered questionnaire. HRBs across various domains were assessed. Validated scales screened for anxiety and depression. Regression analyses determined associations between mental health and HRBs.

**Results:**

Low physical activity was prevalent, with only 10.7% exercising ≥5 times/week. Inadequate fruit/vegetable intake (1–2 servings/day) was reported by 79.9% students. Short sleep duration (5–6 h) on weeknights (51.2%) was common. More than three quarter (76.5%) of students met the screening cutoff score for HRBs. The study highlighted that students with addiction, depression and anxiety are more likely to exhibit HRBs (*p* < 0.01).

**Conclusion:**

Multiple HRBs were highly prevalent among the students. A considerable proportion demonstrated a clustering of risky lifestyle factors, which were linked to poor mental health. Interventions should address academic burden, promote positive health behaviors, and target mental well‐being in this vulnerable group.

## 1 INTRODUCTION

Adolescents and young adults often engage in risky behaviours as they learn to independently navigate the world around them. Such risky or unhealthy behaviours can include smoking, drinking alcohol, not getting enough sleep, and not eating a healthy diet. Indeed, such behaviours are major risk factors for non‐communicable diseases (NCDs).^1^, ^2^

The majority of unhealthy behaviors such as smoking, drinking alcohol, not getting enough sleep, and not eating a healthy diet, are all the major risk factors for NCDs.^3^ Even though these behaviors are common among university students in many parts of the world, they are all things that people can change to improve their health.^4^, ^5^, ^6^

University students face many challenges, such as living away from home, adjusting to a new and diverse environment, trying new types of foods, living independently, making new friends, maintaining their independence, coping with higher‐level studies, and dealing with academic stress.^7^

Adolescence and young adulthood are crucial stages in laying the foundations of health behaviors and healthy lifestyle.^8^ Simultaneously, they equally represent a sensitive period when health risk behaviors (HRBs) can develop.^8^ Particularly, the latter not only contribute to leading causes of morbidity and mortality among young people but also correlate with brain development, cognitive functioning, and academic performance.^8^, ^9^

Previous studies confirmed the correlation between diverse HRBs and self‐perception regarding academic achievement during adolescence.^8^ These include irregular breakfast consumption, insufficient physical activity, excessive screen time exposure, intentional and unintentional injury, and substance abuse, such as smoking.^8^ Furthermore, HRBs established at this age correlate with various health outcomes later in life, even in adulthood.^10^

University students, including those in healthcare fields, are not immune to these risks.^4^, ^5^ We might assume that healthcare students, being future medical professionals, lead healthy lives. However, research indicates that they might partake in behaviors that pose health risks.^11^, ^12^, ^13^ Despite their knowledge of health, medical and nursing students may engage in behaviors that pose health risks.^14^ This paradox underscores the complex interplay between knowledge, behavior, and environmental factors that influence health choices during this crucial developmental period.^6^, ^8^

Mental health issues, including suicidal ideation, are important HRBs among medical and nursing students.^15^ A study conducted in Nepal, found that lifetime suicidal ideation was present in 20.6% of medical students and 13.95% of nursing students.^15^ Academic performance dissatisfaction, parental demands, and psychiatric disorders were associated with higher odds of suicidal ideation among these students.^15^

Given the limited research on HRBs among medical and nursing students in Nepal specifically,^16^ this study aims to fill this gap by examining the prevalence and factors associated with these behaviors. By understanding the specific challenges and risk factors faced by these students, interventions can be developed to promote healthier behaviors and improve overall well‐being.

## 2 METHODOLOGY

### 2.1 Study design and settings

This cross‐sectional study was conducted among medical and nursing students of Lumbini Medical College, Palpa, Nepal, using a self‐administered questionnaire in late August to early September 2023. Nepal is a small country in south‐east Asia landlocked between India and China. It is a low‐middle income country with a majority of Hindu population. Lumbini Medical College, almost 315 km from the capital city Kathmandu, is located in the hilly district of Lumbini province in the western region. This medical college is affiliated to Kathmandu University. It enrolls 100 undergraduate and 24 postgraduate medical students each year. Besides, 40 students are enrolled in the undergraduate nursing program each year.

### 2.2 Sample size

The sample size was calculated assuming a 50% prevalence of HRBs in the population, with a 95% confidence interval and a margin of error set at 5%. The prevalence of 50% was considered to achieve a larger sample size.

The minimum required sample size was calculated to be 384. Additionally, assuming a 5% non‐response rate, the final sample size was determined to be 403 after rounding off.

### 2.3 Study instruments

Data were collected using a structured, self‐administered questionnaire developed by the researchers after an extensive review of the literature (Supporting Information File S1). The questionnaire consisted of four sections:

1. Sociodemographic information
2. Questions on HRBs such as smoking, alcohol use, substance use, physical inactivity, and unhealthy diet
3. Mental health status (measured with the GAD‐7 anxiety scale and the PHQ‐9 depression scale)
4. Factors associated with HRBs, such as stress, peer pressure, and academic performance.

### 2.4 Validity and reliability of instrument

While generating the questionnaire, several measures were used to ensure its reliability and validity. First of all, the questionnaire was content‐wise validated through examining the existing literature and selecting the key constructs. The theoretical frameworks were also located, and the questions to be used to obtain the necessary data were developed. Here, the questions were also tested by the research team to be eligible and perceived by the target population. Construct validity was reached due to the fact that the sections of the instruments measured the constructs evident within the theories of the specific field of study. Finally, the reliability of the questionnaire was established by using the standardized scales: GAD‐7 and PHQ‐9, which have already been tested. We followed the design developed based on the existing sources, and tried to come up with a valid and reliable instrument although it was not pre‐tested. GAD‐7 and PHQ‐9 are the questionnaire‐based tools, designed to assess the severity of anxiety and screening of depression, respectively.^17^, ^18^ The questions are based on the symptoms they have been experiencing in the last 2 weeks. These questionnaires have been validated in the Nepali population.^19^

We followed the design developed based on the existing sources, and tried to come up with a valid and reliable instrument although it was not pre‐tested.

### 2.5 Data collection procedure

The questions were typed into Google form and all authors reviewed it for errors. A convenience sampling technique was utilized where the undergraduate students from medical and nursing school were enrolled. The survey link was then shared with the class representatives of both schools with a request to distribute it through digital platforms such as WhatsApp, Viber, or Facebook messenger to all the students in their respective classes. The first part of the questionnaire consisted of a section where the study objective was detailed, following which there was a section for providing informed consent for participation. It was clearly stated that participation was voluntary, and that no gift (financial or material) would be provided for the completion of the questionnaire. The actual questionnaire was made accessible only to those students who provided their informed consent. For non‐consenting students, the survey was closed, and an incomplete form was submitted. No identifying information such as name or email address was collected. The study included all consenting students studying at Lumbini Medical College and excluded past students, volunteer students, visiting or exchange students. The link was made active on August 27, 2023 and the response was checked every day at 5 p.m. Once the desired sample size was reached on September 2, 2023, the link was disabled to collect any further responses.

### 2.6 Data analysis

All the responses collected were downloaded as a Microsoft Excel Worksheet and then imported to SPSS version 21 and analyzed. Descriptive statistics such as frequencies and percentages were calculated. We then scored the items in the questionnaire in a way that healthy behavior scored the highest points and HRBs received the least points (Supporting Information File S1). The maximum score one could get was 56 if the student practiced healthy behavior. The minimum score was observed to be 25 and the maximum was 49 with a mean of 39.4 and a standard deviation of 4.0. To identify the highest risk group, we selected individuals with scores below one standard deviation above the mean. One standard deviation above the mean of 39.4 is 43.4 (since SD is 4.0). Rounding this up, we could select a cutoff score of 43 and those scoring less than this score were deemed to have HRB. The data were evenly distributed. Linear regression analysis was done to identify factors associated with HRBs. Linear regression is suitable for modeling relationships with continuous dependent variables.

### 2.7 Ethical approval

Ethical approval was obtained from the Institutional Review Committee of the Lumbini Medical College (IRC‐LMC‐05/Q‐23).

## 3 RESULTS

There were 416 responses received of which 4 were from nonconsenting students, thus excluded. The study collected data from 412 medical and nursing students of whom 233 were women (56.6%) and 179 men (43.4%) with a male‐female ratio of 1:1.30. The majority were medical students (87.6%) and unmarried (99.8%). Most were Hindu (94.2%). The demographic details of the participants are depicted in Table 1.

**Table 1:** Socio‐demographic distribution of the respondents.

Variables	Frequency *N* (%)
Sex	
Male	179 (43.4)
Female	233 (56.6)
Type of student	
Medical student	361 (87.6)
Nursing student	51 (12.4)
Marital status	
Unmarried	411 (99.8)
Married	1 (0.2)
Religion	
Hindu	390 (94.7)
Buddhist	16 (3.9)
Islam	4 (1.0)
Christian	2 (0.5)
Year of study	
First	187 (45.4)
Second	92 (22.3)
Third	79 (19.2)
Fourth	44 (10.7)
Final or rotatory internship (medical students only)	10 (2.4)

Regarding physical activity, only 10.7% engaged in moderate to vigorous activity 5 or more times per week. For fruit/vegetable intake, 79.9% had 1–2 servings per day. For sleep, 51.2% got 5–6 h on weeknights. When the students were asked about the source of stress, a high majority of students (90.5%) reported academic workload to be a major stressor, followed by financial pressure (23.1%) and personal relationships (21.8%). Table 2 summarizes the frequency data on various HRBs like physical activity, diet, sleep, substance use, stress, and coping techniques.

**Table 2:** Description on health risk behavior among participants.

Items in the questionnaire	Frequency *N* (%)	95% CI
How often do you engage in moderate to vigorous physical activity per week? (e.g., brisk walking, jogging, gym workouts)		
Never	35 (8.5)	3.13 (3.02–3.24)
Less than once a week	84 (20.4)	
1–2 times a week	130 (31.6)	
3–4 times a week	119 (28.9)	
Five or more times a week	44 (10.7)	
On average, how many servings of fruits and vegetables do you consume per day?		
None	28 (6.8)	2.08 (2.03–2.12)
1–2 servings	329 (79.9)	
3–4 servings	53 (12.9)	
5 or more servings	2 (0.5)	
How many hours of sleep do you typically get on a weeknight?		
Less than 5 h	20 (4.9)	2.42 (2.36–2.48)
5–6 h	211 (51.2)	
7–8 h	168 (40.8)	
More than 8 h	13 (3.2)	
Have you used any of the following substances in the past 12 months? (Alcohol, tobacco, marijuana, prescription drugs without medical supervision or any other)		
Three or more	2 (0.5)	4.50 (4.43–4.56)
Any three	7 (1.7)	
Any two	9 (2.2)	
Any one	160 (38.8)	
None	234 (56.8)	
What are the primary sources of stress in your life? (Academic workload, clinical responsibilities, financial pressure, personal relationships, familial issues, or any other)		
Three or more	6 (1.5)	3.55 (3.48–3.62)
Any three	35 (8.5)	
Any two	99 (24.0)	
Any one	270 (65.5)	
None	2 (0.5)	
How do you typically cope with stress? (Exercise, socializing with friends/family, relaxation techniques [e.g., deep breathing, meditation], unhealthy coping mechanisms [e.g., excessive eating, substance use], or any other)		
Unhealthy coping technique	26 (6.3)	3.05 (2.96–3.13)
Any healthy and/or unhealthy coping technique	45 (10.9)	
Any healthy coping technique	253 (61.4)	
Two healthy coping techniques	60 (14.6)	
Three or more healthy coping techniques	28 (6.8)	

Abbreviation: CI, confidence interval.

Using validated scales, 41.5% had minimal depression, 33.5% had mild depression, and 16.3% had moderate depression. For anxiety, 34.7% had minimal anxiety, 44.9% had mild anxiety, and 15.8% had moderate anxiety. Academic performance was generally high, with 51.5% having a GPA of 3.0–3.5 and 17.5% above 3.5. Most (96.8%) were aware of preventive health measures, although only 6.8% frequently participated in health promotion activities. Table 3 provides a summary of the prevalence of depression, anxiety, burnout symptoms, healthcare utilization, and academic performance. When the students were screened with the cut‐off score for HRB, there were 97 students (23.5%) scoring 43 or more engaging in healthier behavior and remaining 315 students (76.5%) indulged in some HRB.

**Table 3:** Distribution of various psychological and academic variables among respondents.

Variables	Frequency *N* (%)
Patient Health Questionnaire (PHQ‐9) Depression Test	
Severe depression	12 (2.9)
Moderately severe depression	24 (5.8)
Moderate depression	67 (16.3)
Mild depression	138 (33.5)
No depression	171 (41.5)
Generalized Anxiety Disorder Assessment (GAD‐7)	
Severe anxiety	19 (4.6)
Moderate anxiety	65 (15.8)
Mild anxiety	185 (44.9)
No or minimal anxiety	143 (34.7)
How frequently do you experience symptoms of burnout?	
Always	13 (3.2)
Often	57 (13.8)
Sometimes	112 (27.2)
Rarely	140 (34.0)
Never	90 (21.8)
How often do you seek healthcare services when needed	
Always	90 (21.8)
Often	108 (26.2)
Sometimes	118 (28.6)
Rarely	79 (19.2)
Never	16 (3.9)
What was your Grade Point Average (GPA) in the last examination you appeared?	
Below 2.0 (Fail: Below 31.99%)	3 (0.7)
2.0–2.5 (Third division: Above 32% below 49.99%)	27 (6.6)
2.5–3.0 (Second division: Above 50% and below 59.99%	98 (23.8)
3.0–3.5 (First division: Above 60% and below 74.99%)	212 (51.5)
Above 3.5 (Distinction: above 75%)	72 (17.5)

Investigating the relationship between “Health risk behavior score” and “PHQ score,” a Pearson correlation analysis was conducted. The results revealed a significant negative correlation between the two variables (*r* = −0.680, *p* < 0.01). This indicates that as healthy behavior increases depressive symptoms decrease and vice versa. The strength of the negative correlation suggests that as “Health risk behavior score” increases, “PHQ score” tends to decrease, implying a moderate to strong inverse relationship between healthy behaviors and depressive symptoms.

### 3.1 Normality checks of the model under study

**Figure 1:**
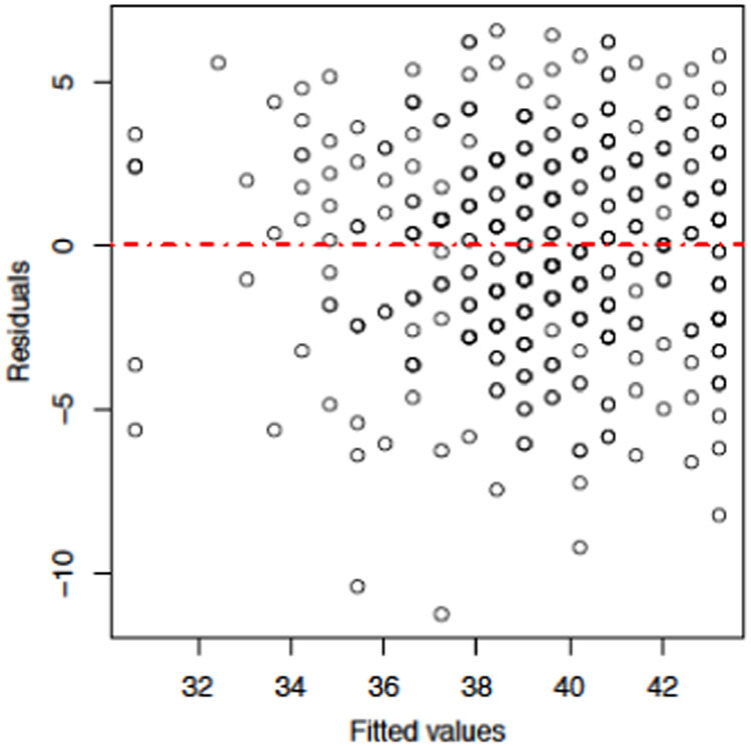
Residual plot of the model under study.

**Figure 2:**
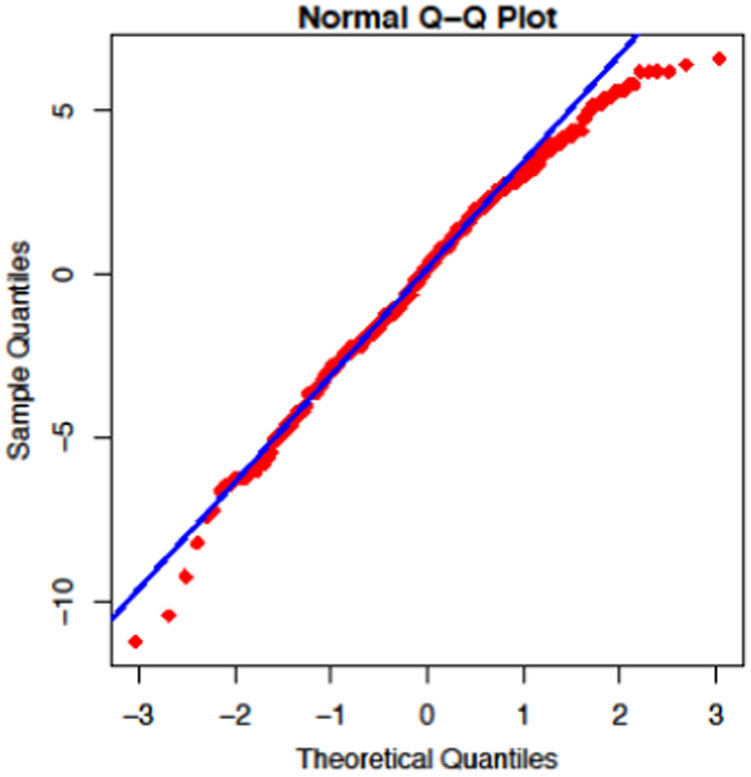
Q‐Q plot for the normality check.

We can check the assumption of linear regression model by using residual analysis and Quantile‐Quantile (Q‐Q) plot. The residual analysis of the model under study are displayed in Figure 1. Similarly, for normality check we have plotted Q‐Q plot as presented in Figure 2. By observing the graphs in both the figures, we are confirmed that our model satisfies the required conditions for the linear regression analysis.

**Figure 3:**
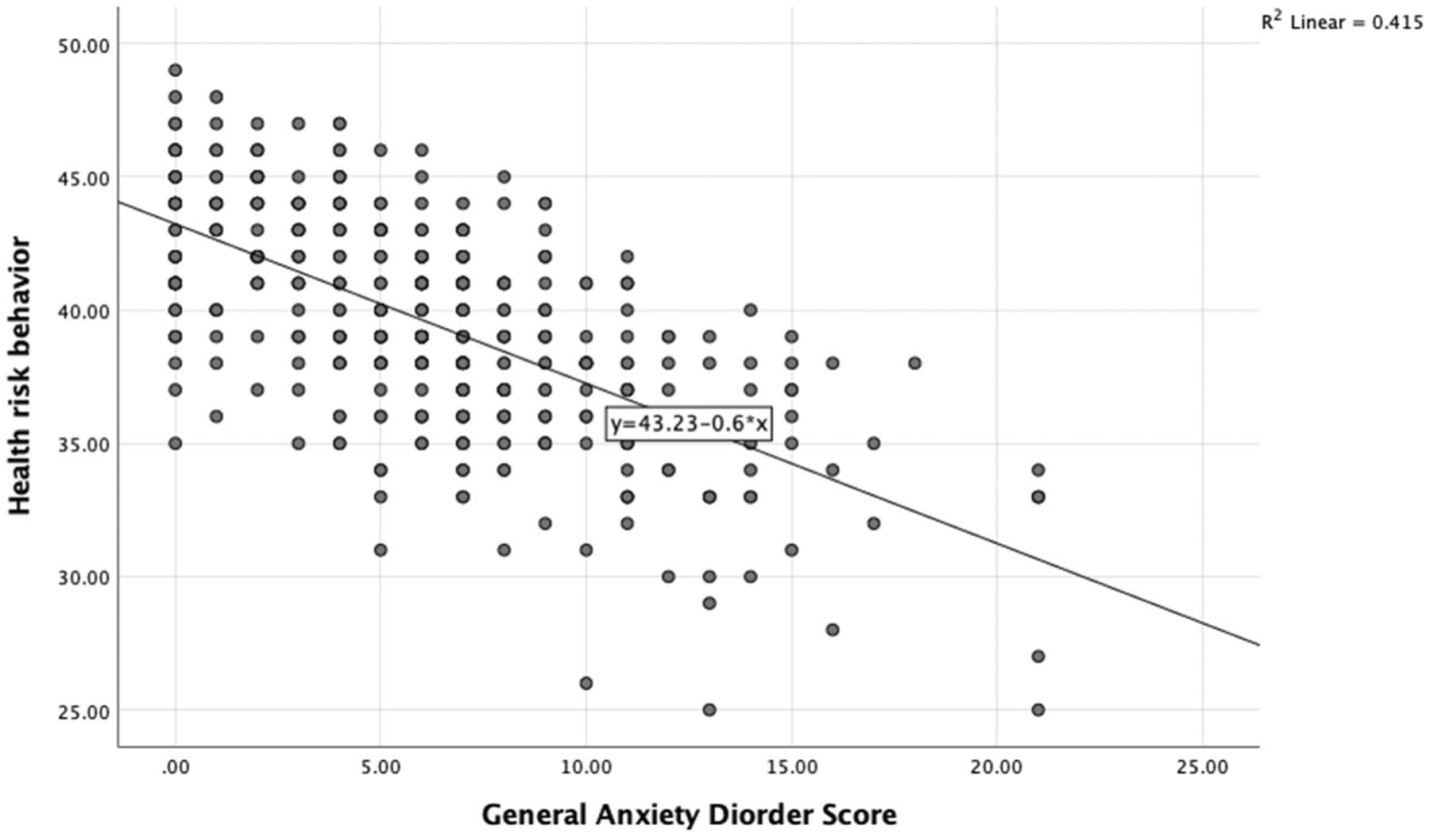
Scatter plots showing a relationship between anxiety score and health risk behavior score.

It is worth mentioning that the scatter plots visually show a noticeable downward trend, suggesting a potential negative correlation between anxiety scores and HRB scores (Figure 3). This trend indicates that as HRB scores increase (indicating healthier behavior), General Anxiety Disorder score tend to decrease. This relationship is further supported by the result of the linear regression analysis, which yields an *R* ^2^ value of 0.415. This *R* ^2^ value signifies that approximately 41.5% of the variance in HRB scores can be explained by GAD scores within the context of the linear regression model. We employed linear regression to test this association, given that our HRB scores ranged from 25 to 49, itself a continuous measure, and we had observed linear pattern in the scatter plots. Before the analysis, we verified the assumptions of linear regression through residual analysis and Q‐Q plots, which confirmed the normality of residuals and homoscedasticity. This approach allowed us to estimate the strength and direction of the relationship between exposures, while allowing for the examination of several predictors simultaneously, aligning with study objective of identifying factors associated with HRB. The resulting regression coefficients provide easily interpretable measures that elucidates how variations in mental health scores relate to changes in HRB scores, thereby providing valuable insights about these associations. This suggests a moderate to strong relationship where healthier behaviors are associated with lower level of anxiety among the students in this study.

Chi‐square tests revealed significant associations between some socio‐demographic factors and HRBs (Table 4). Age showed a weak but significant association (*p* = 0.033), with younger participants (17–20 years) exhibiting the highest prevalence of HRBs (88.0%). Sex had a highly significant association (*p* = 0.0001) with HRBs. Females exhibited a higher prevalence of HRBs (88.8%) compared to males (74.9%). Type of student (*p* = 0.468) and religion (*p* = 0.234) were not significantly associated with HRBs.

**Table 4:** Chi‐square test to test the independence of attributes of HRB against various socio‐demographic variables.

Variables	Health risk behavior		*p*‐value
	Present	None	
Age			0.033*
17–20	117 (88.0)	16 (12.0)	
21–24	212 (81.2)	49 (18.8)	
≥25	11 (64.7)	6 (35.3)	
Sex			<0.001*
Male	134 (74.9)	45 (25.1)	
Female	207 (88.8)	36 (11.2)	
Type of student			0.47
Medical student	298 (82.5)	63 (17.5)	
Nursing student	43 (84.3)	8 (15.7)	
Religion			0.23
Hindu	320 (82.3)	69 (17.7)	
Other than Hindu	20 (90.9)	2 (9.1)	

*p < 0.05.

## 4 DISCUSSION

According to the results of the present study, almost 77% of students indulged in risky behaviors across all the measured dimensions, including physical activity, sleep, dietary habits, substance use, and mental well‐being.

Being inactive is typical of medical students due to their busy schedules and academic pressure which leaves little time for them to exercise.^6^, ^20^ Furthermore, their strong emphasis on academic careers often sparks a need to protect their grades, which is why they ignore the importance of their fitness.^21^ The sedentary character of their studies, entailing extended periods seated at desks, also does not encourage active lifestyles.^22^ When facing academic pressures, students often opt for convenient yet unhealthy dietary choices and frequently forego morning workouts.^22^ Indeed, many lack awareness of both the physical and psychological benefits of regular exercise.^23^

We observed that almost 90% of the students did not engage in moderate to vigorous physical activity. Physical activity affects overall quality of life.^24^ In a study conducted in the same region of Palpa in Nepal, it was noticed that almost two‐thirds of school students did not participate regularly in physical activity, and girls were more sedentary than boys.^25^ Physical inactivity among college students is a common phenomenon observed in various studies around the world, such as Brazil, Canada, India, Thailand, Poland, and the United Kingdom.^20^, ^22^, ^23^, ^26^, ^27^, ^28^, ^29^

Grhouz et al., in their study, observed that a lack of physical activity among Indian college students was linked to sleep disorders and mental health problems.^28^ Notably, we also observed the symptoms of anxiety and depression being more common in our study population, with over 60% of students screening positive for at least mild anxiety and more than 50% screening positive for mild depression. In Cyprus, high‐intensity physical activity was associated with happiness among female medical students in the first year.^21^

More than half of the students reported short sleep duration on weeknights, likely driven by academic pressure. The effects of sleep loss on students’ academic performance, GPA, mood, and learning and memory have already been established.^30^ This finding aligns with a Nepali study that demonstrated poor sleep quality associated with poor academic performance and depression in undergraduate students.^31^

A systematic review and meta‐analysis revealed several HRBs associated with poorer mental health outcomes among medical students during the COVID‐19 pandemic.^32^ Unhealthy lifestyles, including low physical activity, increased substance use, and irregular diet and sleep patterns, were linked to negative mental health effects.^32^ Problematic smartphone or internet use and excessive screen time were also identified as risk factors.^32^ Dissatisfaction with online learning and concerns about educational impairment due to the pandemic were additional risk factors.^32^ Economic instability was also associated with poorer mental health outcomes.^32^

Unhealthy eating habits were also found to be prevalent in our study, which aligns with the results of a survey conducted in Nepal.^33^ Almost all Nepali people do not meet the recommended five servings of fruits and vegetables per day.^33^ This was also fully depicted in our study, where unhealthy eating habits were found to be prevalent among students. It is believed that the lack of food options in cafeterias leads people to choose junk food.^34^

Additionally, our study shows that more than a quarter of students reported engaging in substance use in the past year, including alcohol misuse, tobacco use, marijuana consumption, or prescription drug misuse. This finding is consistent with a study that investigated substance use among Nepali healthcare students.^35^ A study conducted among Nepali medical and nursing students found that substance use (smoking, alcohol consumption) was associated with suicidal ideation in some cases, particularly among nursing students.^15^ A web‐based survey conducted among Iranian university students revealed that tobacco use, alcohol consumption, high‐risk sexual behaviors and drug abuse are prevalent.^11^ Furthermore, a multinational study involving nursing students from Belgium, Brazil, France, and Spain revealed that one‐third of participants had used drugs like cannabis, cocaine and ecstasy (amphetamines) at some point in their lives.^36^ In contrast to these findings, our study indicated that nursing students mainly used over‐the‐counter drugs and prescription medications without supervision, rather than illicit substances. The use of substances poses health risks that can be mitigated through support and counseling services. Many students do not share their mental health problems due to stigma around mental illness in Nepal, which could act as a barrier to addressing HRB.^15^ According to the University of Memphis School of Nursing, it is suggested that healthcare students should address substance addiction through measures such as prevention, education, identification, evaluation, and referral for treatment.^37^ Instead of being dismissed, this alternative approach ensures that students receive proper care and support, preventing them from feeling neglected or ashamed and reducing the risk of incidents, like overdosing or self‐harm.^37^

The findings of this study make several novel contributions to our understanding of HRB among medical and nursing students in Nepal. Although similar studies have been conducted in other countries, it might differ from other Western or Asian countries according to the cultural context of this Hindu‐dominated Nepali population. Furthermore, data on this particular population in Nepal were previously limited. Our comprehensive assessment of multiple HRBs along with mental health indicators provides a holistic view that is rare in the literature.

Perhaps most significant is the finding that over 76% of students exhibited multiple HRBs concurrently. This clustering of risk factors represents a significant public health concern that may have been underappreciated. The strong association we found between poor mental health and increased HRB provides new evidence on the interconnection of these issues in Nepali healthcare students. Additionally, our quantification of academic pressure as the primary stressor offers valuable local data.

The results of this study allow concluding that there is a need for institutional policies and interventions that contribute to encouraging positive health behaviors among medical and nursing students. More specifically, health promotion and wellness programs should be included in the academic curriculum, and mental health support to students should be made more accessible and appropriate. Additionally, as academic pressure is the central agent of stress in the lives of students, promoting a better work‐life balance among this population can involve teaching students how to schedule and manage their time effectively. Finally, to ensure that the intervention designed to reduce HRBs in college‐goers is efficient and ongoing, one will need to embed a range of dimensions within it: the role of educational organizations and institutions, healthcare providers, the policy‐making arena, and students’ organization.

One of the powerful aspects of the study is that it utilizes a large sample of 412 medical and nursing students. In such a way, a high statistical power ensues, which allows for precise and reliable estimation of prevalence rates and identification of associations. Additionally, the valid psychometric properties of measures used to assess mental health variables, such as the GAD‐7, and depression, as measured by PHQ‐9, contribute to the credibility of results. Furthermore, the comprehensive assessment of both multiple domains of HRB, such as physical activity, diet, sleep, substance use, and mental health, and indicators offers a broad view of the phenomenon. In this way, the study of associations of mental health indicators with HRBs contributes to identifying the possible clustering of risk behaviors. Finally, the study focuses on a poorly studied but critical group of future healthcare providers, which increased the relevance of the study. The results stratification by student group helps to identify the possible differences in risk profiles. The use of online data collection was a convenient and efficient method for the student population and addressed some biases associated with in‐person interviews. Investigating of various factors, such as academic stress, and coping mechanisms, allows for a more detailed examination of the issue.

Limitations of the present study were that the study was conducted at a single medical college and used a convenience sampling approach. As such the results may not be generalizable across other student populations or geographic regions, and further multi‐center studies using representative sampling techniques are required to enhance the generalizability of the results.

While this study examined several critical HRBs, future work might consider additional dimensions of behavior that are more salient to healthcare student populations. In practice, it may be useful to explore the relationships between excessive screen time and its link with problematic social media use or gaming habits in accessing the health and well‐being of medical and nursing students. This has led these digital behaviors to be ubiquitous among the young adults and are associated with physical inactivity, sleep disturbances, and mental health issues. Quantifying the prevalence of these behaviors and their associations with academic performance, stress levels, and overall health might provide some critical insights. Such studies may be used to develop targeted interventions that encourage balanced and healthy technology use within healthcare student populations, which could theoretically increase healthcare students own well‐being, while also equipping them with knowledge around these issues in order counsel future patients.

## 5 CONCLUSION

This cross‐sectional study conducted among medical and nursing undergraduates at Lumbini Medical College has revealed an alarmingly high prevalence of multiple HRBs, such as physical inactivity, unhealthy eating habits, sleeping less than 6 h a day, tobacco/alcohol/substance use, and anxiety/depression. Nearly one‐quarter of the participants met the screening cutoff score, indicating participation in adverse health behaviors. Their poor mental health status was strongly associated with the exhibition of these risky lifestyle factors. Academic pressure was considerably high among various stressors. Multiple HRBs have now become widely prevalent in developing countries, but the risk factor profiles have not been extensively studied yet.

This vulnerable population should be the concern of every educational institution, and probable interventions to increase good health behaviors should be focused on them. Thus, the academic load should be reduced and good habits, such as regular physical activity and sleep, should be increased. Students should also be taught about healthy dieting, and the opportunities for using counseling support services. It is crucial for the approaches to focus on what is making these occurrences happen, so that more prevention‐based interventions can be developed during research. A collaborative approach of all stakeholders is required to foster the good physical and mental health of prospective healthcare providers.

## AUTHOR CONTRIBUTIONS

**Samata Nepal**: Conceptualization; formal analysis; data curation; methodology; writing—original draft; writing—review and editing. **Alok Atreya**: Data curation; investigation; writing—original draft; writing—review and editing. **Kishor Adhikari**: Methodology; formal analysis; writing—review and editing. **Bhumika Acharya**: Conceptualization; data curation; writing—original draft. **Ritesh G. Menezes**: Project administration; supervision; resources; writing—review and editing. **Laxmi Prasad Sapkota**: Methodology; formal analysis; writing—review and editing.

## CONFLICT OF INTEREST STATEMENT

The authors declare no conflicts of interest.

## TRANSPARENCY STATEMENT

The lead author Samata Nepal affirms that this manuscript is an honest, accurate, and transparent account of the study being reported; that no important aspects of the study have been omitted; and that any discrepancies from the study as planned (and, if relevant, registered) have been explained.

## Supporting information

Supporting information.

## Data Availability

The datasets generated during and/or analyzed during the current study are freely available from https://osf.io/f2dvr/.
