# Supplementary material for: Health risk behaviors among medical and nursing students of Lumbini Medical College, Nepal: A cross‐sectional study
Source: Health Sci Rep. 2024 Oct 16;7(10):e70140. doi: 10.1002/hsr2.70140 (PMC11483537; doi:10.1002/hsr2.70140)
Supplement: Supplementary file 1 — Supporting information. [file HSR2-7-e70140-s001.docx]

**Proforma and the scoring**

Title: **Health Risk Behaviors among Medical and Nursing Students of Lumbini Medical College**

**Section 1: Demographic Information**

1.Sex: Male / Female / Other (Please specify: ____)

2. Age: ____

3. School: School of Medicine/ School of Nursing

Year of Study: 1st / 2nd / 3rd / 4th / 5^th^ or rotatory internship (Medical students)

4. Marital Status: Single / Married / Other (Please specify: ____)

5. Religion: ____

**Section 2: Lifestyle and Health Habits**

6. How often do you engage in moderate to vigorous physical activity per week? (e.g., brisk walking, jogging, gym workouts)

- Never (1 point)

- Less than once a week (2 points)

- 1-2 times a week (3 points)

- 3-4 times a week (4 points)

- 5 or more times a week (5 points)

7. On average, how many servings of fruits and vegetables do you consume per day?

- None (1 point)

- 1-2 servings (2 points)

- 3-4 servings (3 points)

- 5 or more servings (4 points)

8. How many hours of sleep do you typically get on a weeknight?

- Less than 5 hours (1 point)

- 5-6 hours (2 points)

- 7-8 hours (3 points)

- More than 8 hours (4 points)

9. Have you used any of the following substances in the past 12 months? (Select all that apply)

- Alcohol

- Tobacco

- Marijuana

- Prescription drugs without medical supervision

- Other (Please specify: ____)

Scoring:

None (5 points)

Any one (4 points)

Any two (3 points)

Any three (2 points)

More than three (1 point)

**Section 3: Stress and Coping Mechanisms**

10. What are the primary sources of stress in your life? (Select all that apply)

- Academic workload

- Clinical responsibilities

- Personal relationships

- Financial pressures

- Other (Please specify: ____)

Scoring:

None (5 points)

Any one (4 points)

Any two (3 points)

Any three (2 points)

Any 4 (1 point)

11. How do you typically cope with stress? (Select all that apply)

- Exercise

- Socializing with friends/family

- Relaxation techniques (e.g., deep breathing, meditation)

- Unhealthy coping mechanisms (e.g., excessive eating, substance use)

- Other (Please specify: ____)

5 points

**Section 4: Mental Health (GAD-7 scale) Level of Anxiety**

Questions 12 to 18

Scoring:

4 points if Minimal Anxiety (0-4 in GAD-7 scale)

3 points if Mild Anxiety (5-9 in GAD-7 scale)

2 points if Moderate Anxiety (10-14 in GAD-7 scale)

1 point if Severe Anxiety (score greater than 15 in GAD-7 scale)

**Section 5: Mental Health** **(PHQ-9 scale) Level of Depression**

Questions 19 to 27

Scoring:

5 points if Minimal or no depression (PHQ-9 score is 0-4)

4 points if Mild depression (PHQ-9 score is 5-9)

3 points if Moderate depression (PHQ-9 score is 10-14)

2 points if Moderately severe depression (PHQ-9 score is 15-19)

1 point if Severe depression (PHQ-9 score is 20-27)

**Section 6: Factors associated with health risk behaviors**

28. How frequently do you experience symptoms of burnout? (Select one)

- Never (5 points)

- Rarely (4 points)

- Sometimes (3 points)

- Often (2 points)

- Always (1 point)

29. What is your current Grade Point Average (GPA)?

- Below 2.0 (1 point)

- 2.0-2.5 (2 points)

- 2.5-3.0 (3 points)

- 3.0-3.5 (4 points)

- Above 3.5 (5 points)

30. On average, how many hours per week do you dedicate to studying?

31. How often do you seek healthcare services when needed?

- Always (5 points)

- Often (4 points)

- Sometimes (3 points)

- Rarely (2 points)

- Never (1 point)

32. Are you aware of preventive measures for common health issues? (e.g., vaccination, regular check-ups)

- Yes

- No

33. How frequently do you engage in health promotion activities (e.g., health campaigns, workshops, seminars)?

- Regularly (4 points)

- Occasionally (3 points)

- Rarely (2 points)

- Never (1 point)

**Section 7: Additional Comments**

34. Is there anything else you would like to share related to health risk behaviors, stress management, or any other relevant topics?
